# Modeling interrelationships between health behaviors in overweight breast cancer survivors: Applying Bayesian networks

**DOI:** 10.1371/journal.pone.0202923

**Published:** 2018-09-04

**Authors:** Selene Xu, Wesley Thompson, Jacqueline Kerr, Suneeta Godbole, Dorothy D. Sears, Ruth Patterson, Loki Natarajan

**Affiliations:** 1 Department of Mathematics, University of California, San Diego, San Diego, California, United States of America; 2 Department of Family Medicine and Public Health, University of California, San Diego, San Diego, California, United States of America; 3 Department of Medicine, University of California, San Diego, San Diego, California, United States of America; Vanderbilt University, UNITED STATES

## Abstract

Obesity and its impact on health is a multifaceted phenomenon encompassing many factors, including demographics, environment, lifestyle, and psychosocial functioning. A systems science approach, investigating these many influences, is needed to capture the complexity and multidimensionality of obesity prevention to improve health. Leveraging baseline data from a unique clinical cohort comprising 333 postmenopausal overweight or obese breast cancer survivors participating in a weight-loss trial, we applied Bayesian networks, a machine learning approach, to infer interrelationships between lifestyle factors (e.g., sleep, physical activity), body mass index (BMI), and health outcomes (biomarkers and self-reported quality of life metrics). We used bootstrap resampling to assess network stability and accuracy, and Bayesian information criteria (BIC) to compare networks. Our results identified important behavioral subnetworks. BMI was the primary pathway linking behavioral factors to glucose regulation and inflammatory markers; the BMI-biomarker link was reproduced in 100% of resampled networks. Sleep quality was a hub impacting mental quality of life and physical health with > 95% resampling reproducibility. Omission of the BMI or sleep links significantly degraded the fit of the networks. Our findings suggest potential mechanistic pathways and useful intervention targets for future trials. Using our models, we can make quantitative predictions about health impacts that would result from targeted, weight loss and/or sleep improvement interventions. Importantly, this work highlights the utility of Bayesian networks in health behaviors research.

## Introduction

Obesity, physical inactivity, and impaired sleep are known risk factors for cardiovascular disease, type 2 diabetes, and cancer [1–8]. Sedentary behavior is also reported to increase risk for chronic disease and mortality [9, 10]. It is recognized that multiple biological pathways, such as glucose regulation and inflammation, are implicated in the link between health behaviors, obesity and chronic disease [11, 12]. Unraveling interrelationships among these factors could elucidate disease mechanisms and inform design of clinical studies.

Modeling multiple correlated factors can be computationally challenging and requires new statistical approaches. Standard regression modeling cannot disentangle these complex associations. Bayesian graphical networks are a novel and powerful approach for examining relationships among multiple correlated variables. These models provide algorithms for discovering and analyzing structure, as well as, an intuitive graphical interface for visualizing multivariate distributions. Bayesian networks were initially developed in computer science and artificial intelligence applications [13, 14], and have since had major impact in biomedicine, ‘omics studies, neurosciences, and more recently in obesity research [15–23]. To our knowledge, no existing obesity studies have examined multiple lifestyle factors simultaneously in conjunction with biomarkers and psychosocial factors in a cancer survivor population.

In this work, we applied probabilistic Bayesian networks [13, 24] to elicit bio-behavioral pathways implicated in obesity and health in breast cancer survivorship. Our sample comprised 333 well-characterized postmenopausal breast cancer survivors with objective physical activity assessments, detailed information on multiple lifestyle factors, clinical characteristics, and a variety of health measures [25]. Using this unique sample, we developed Bayesian networks to model inter-relationships between health behaviors (sleep, physical activity), body mass index (BMI), circulating biomarkers of inflammation (C-reactive protein [CRP]) and glucose regulation (fasting insulin), and mental and physical quality of life.

### Brief description of Bayes network methodology

A Bayesian network is a probabilistic directed acyclic graph. Random variables are depicted as nodes on the graph, and edges between nodes represent dependencies (e.g., partial correlations) between these variables. If there is a directed link (arrow) from node A to node B, then A is termed the “parent” and B the “child”. Each node has an associated distribution function that takes as input a set of values for the node's parent variables and gives the probability of the variable represented by the node. The presence of an edge or path between two variables indicates a non-zero partial correlation between the two variables.

Fitting a Bayesian network requires (i) learning its structure, namely which nodes in the graph are connected, and (ii) estimating parameters associated with conditional probabilities. Specifically, let ***X*** comprise the set of variables X_i_ (e.g., X_1_ = physical activity, X_2_ = sleep quality, X_3_ = BMI, X_4_ = insulin level, etc.) and *M* be a Bayesian network on ***X***, comprising a directed acyclic graph of edges between variables in ***X***. The model *M* encodes conditional independencies that imply a factoring of the joint probability distribution p(***X***) of ***X*** [13]:

$$\mathrm{Pr}\left(X|M\right)=\Pi \;\mathrm{Pr}\left(X_{i}|pa(X_{i})\right)$$ (1)

where π represents the product of conditional probability distributions, and pa(*X*_i_) denotes variables (“parents”) in ***X*** with arrows leading into *X*_i_. The structure of the graph *M* can be learned by implementing constraint-based, search-score, and hybrid algorithms [26]. For a given graph *M*, Pr(*X*_i_ | pa(*X*_i_)) represents a local probability distribution, and its parameters **ß** can be estimated by regression methods, using multivariate Gaussian distributions (after appropriate transformation if needed) or non-parametric approaches for continuous variables and multinomial distributions for categorical variables [24, 27]. Thus, dependencies and (conditional) independencies between sets of variables can be derived from a Bayesian network analysis.

The notion of a Markov blanket of a Bayesian network can be used to identify sets of variables that are (conditionally) independent (i.e., uncorrelated). The Markov blanket for a node V in a Bayesian network is the set of nodes composed of V’s parents, children and its children’s other parents. Conditional on its Markov blanket, a node V is independent of other nodes in the network [24]. Thus the Markov blanket gives an indication of the set of variables that have direct associations with V. Other variables not in the Markov blanket, may be associated with V, but only indirectly through the variables in the Markov blanket. This is what would be expected under a causal interpretation, although one must be cautious in this interpretation for observational data.

## Materials and methods

### Ethics statement

This was a secondary data analysis of the “Reach for Health” clinical trial carried out at the University of California, San Diego (UCSD). The original study was approved by the UCSD IRB board, project #101977. All subjects in the Reach for Health study provided written consent. The National Institutes of Health ClinicalTrials.gov identifier is NCT01302379.

### Study sample and measures

#### Study sample

Our study sample comprised 333 early-stage breast cancer survivors enrolled in a weight-loss intervention. The study protocol and design have been previously published [25]. Briefly, the study enrolled breast cancer survivors, who were postmenopausal at cancer diagnosis, were either overweight or obese at study entry, and had completed primary breast cancer treatment (surgery with or without chemotherapy and radiation). 83% were white; 11% were Hispanic. More information on demographics, lifestyle, clinical factors, coping, sleep, mood, physical factors, and biomarkers is provided in Table 1. The current analysis used baseline information to develop network models.

**Table 1:** Characteristics of the Reach for Health cohort of overweight postmenopausal breast cancer survivors (N = 333).

	Variables (Nodes)	
Demographics & Lifestyle	Age (years) Mean (SD)	63 (6.9)
	Education (% with college or higher degree)	51%
	Smoke (% who ever smoked)	45%
	Alcohol (drinks/month) Median	4
Clinical Factors	Tumor Stage %	48% Stage1 35% Stage2 17% Stage3
	Years from cancer diagnosis to study entry (years) Mean (SD)	2.7 (2.0)
	Estrogen Receptor (% Positive)	85.0%
	Progesterone Receptor (% Positive)	71.8%
Cancer Treatment	Treatment type (%)	53.2% chemotherapy 72.1% radiation 76.9% endocrine 13.8% immunotherapy
Coping	Monitor-blunter (MB) Mean (SD)	4.2 (3.5)
Neighborhood	NEWS scale Mean (SD)	3.1 (1.7)
Health	Insomnia %	28.8% Yes
	Depression %	40.8% Yes
	Arthritis %	56.4% Yes
	QOLp (SF-36) Mean (SD)	66.2 (18.7)
	QOLm (SF-36) Mean (SD)	73.6 (18.4)
	Insulin^+^ (pg/mL) Median (25^th^, 75^th^)-%ile	463.8 (335.6, 667.0) pg/mL
	CRP (mg/l) Median (25^th^, 75^th^)-%ile	3.1 (1.5, 6.5) mg/l
Health Behaviors	Total Physical Activity (PA) (counts/min/day) Mean (SD)	273.1(108.6)
	MVPA^++^ min/day Mean (SD)	17.5 (17.3) min/day
	Sedentary time min/d Mean (SD)	471(111) min/day
	BMI (kg/m^2^) Mean (SD)	31.1(4.9) kg/m^2^
	Sleep Disturbance Score (Sleep1) Mean (SD)	50.5 (8.8)
	Sleep Impairment Score (Sleep2) Mean (SD)	46.9 (9.0)

^+^ To convert insulin pg/mL to pmol/L multiply by 0.172

^++^Weartime adjusted

#### Measures

We obtained participants’ medical records including tumor characteristics (*Cancer Stage*, *hormone receptor status*) and years from cancer diagnosis to study entry (*YrsDXRND*). During clinic visits, participants’ height and weight were measured and used to calculate BMI. Physical activity (PA) and sedentary behavior (SB) were determined by objective 7-day, minute-level triaxial accelerometer counts. Specifically, PA was the average (across days) of total counts per minute per day, thus representing a measure that captured total volume of activity; moderate vigorous physical activity (*MVPA*) was the average of minutes per day with counts ≥ 1952; SB was the average of minutes per day with counts < 100. Accelerometer-derived measures were adjusted for device wear-time. Demographic information and other study measures were obtained through self-report or questionnaires. The Neighborhood Environment Index (*Neighborhood*) derived from the NEWS scale [28] was used to measure walkability. It has a range from 0 to 6, with higher scores indicating more walkable neighborhood. Sleep quality was evaluated based on the PROMIS scale [29]. In the current analysis, we used two subscales, the sleep disturbance (*sleep1*), and the sleep impairment (*sleep2*) subscales. These subscales were normed to mean 50 with standard deviation of 10. Higher scores indicated worse sleep. Quality of life assessment, both mental (QOLm) and physical (QOLp), used the SF-36 scale [30]. QOLm and QOlp scores from 0 to 100, with higher scores reflecting better quality of life. The Monitor-Blunter (MB) scale assessed participants’ coping mechanism. It ranged from -16 to 16, with higher scores indicating more monitor than blunter. Fasting plasma CRP and insulin concentrations were measured using immune-based assays (Meso Scale Discovery), as described previously [25].

### Statistical methods

We fit a Bayesian network to examine multivariate relationships between demographics, clinical factors, health behaviors and health outcomes. We disallowed implausible edge directions while learning the network structure. Specifically, we disallowed QOLp and QOLm to be the parent nodes of any other variable in the network; and we disallowed age, education, cancer stage, years between diagnosis and study entry, and neighborhood to be the child nodes of any other variable. We applied bootstrap resampling to learn a set of 500 network structures. We then averaged these networks in an attempt to reduce the impact of locally optimal (but globally suboptimal) networks on learning and inference. The averaged network is a more robust model with better predictive performance than choosing a single, high-scoring network [24]. To quantify stability of inferred edges, we computed *arc strength* and *direction strength*. Arc strength was calculated as the frequency of an edge occurring between two variables across the 500 bootstrapped network structures; similarly, directional strength was assessed as the frequency of the observed direction re-occurring in the set of learned network structures in which the relevant edge occurred. The averaged network was created using the arcs whose strength exceeded a threshold, which was computed by searching for the arc set “closest” to the arc strength computed from the original data [24]. Conditional independencies were inferred using Markov blankets and related Bayesian network theory.

We used Bayesian information criteria (BIC) and posterior model probabilities to compare fit of candidate networks. The BIC was computed as logLik(M)– 0.5*k*log (n), where logLik(M) is the log-likelihood of model M, k is the number of parameters in M, n is the sample-size. This is the classic definition rescaled by -2; hence, in our calculations, higher BIC scores indicate better fit. We also calculated the Bayes factor, which is the ratio of the posterior probabilities (given the observed data) of the first to the second model, as another metric to compare the two models. The log of the Bayes factor can be approximated as the difference in the BIC scores as defined above [31]. Finally, we used logic sampling [24] to study how small perturbations got propagated through the network. In other words, using Monte Carlo simulations, we evaluated how changes in one part of the network could influence distributions in another part of the network, and thus potentially predict the impact of manipulating specific variables. Biomarkers were log-transformed to better approximate Gaussian assumptions. Models were fitted using the R package bnlearn [32].

## Results

### Decomposition of probability distribution

**Fig 1:**
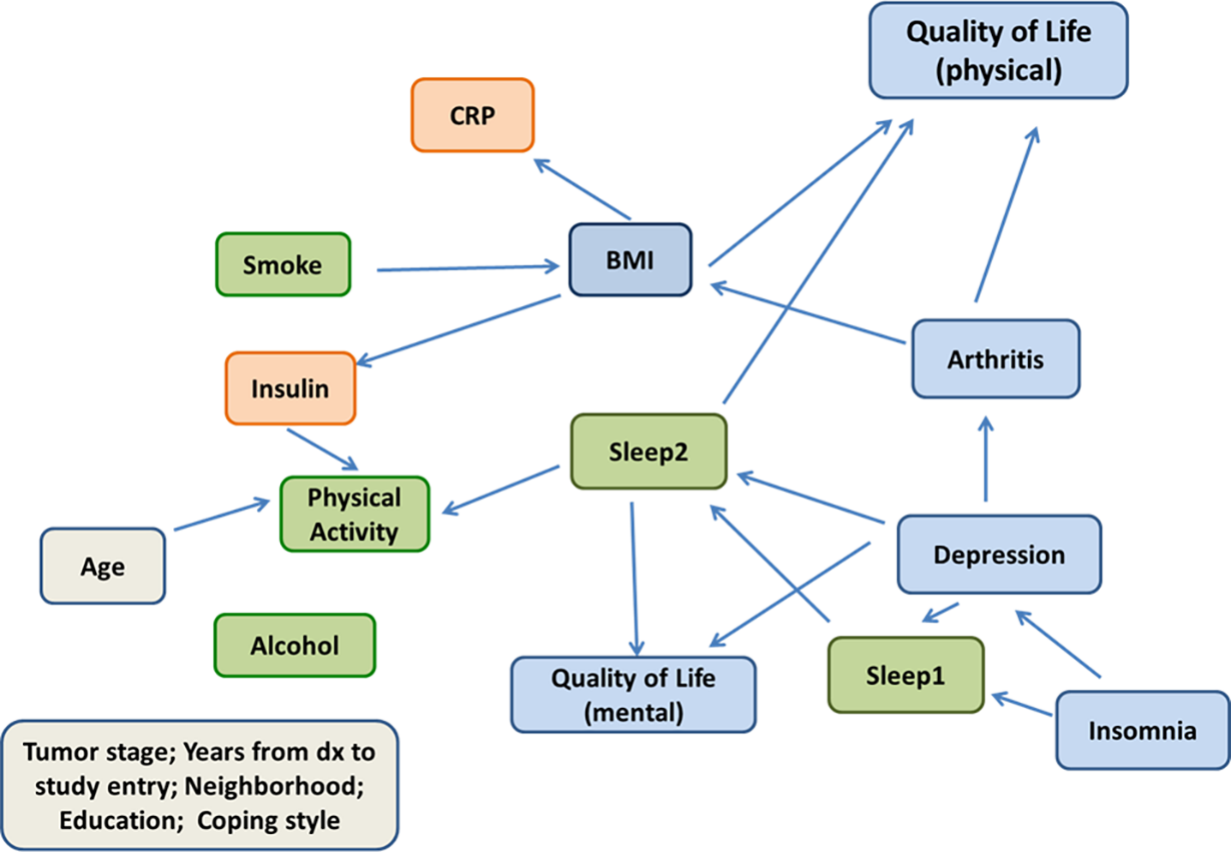
Bayesian network of total physical activity, other lifestyle factors and health in the Reach for Health cohort. Lifestyle factors (green), biomarkers (orange), and physical and mental health outcomes (blue).

The fitted network is shown in Fig 1. From the network analysis, we can obtain the joint probability distribution of all the variables as a product of conditional distributions. In our application, we obtained

$$\begin{array}{l} P\left(x\right)\\ =P\left(Tumor\;Stage\right)\cdot P\left(Years\;diagnosis\;to\;study\;entry\right)\cdot P\left(Neighborhood\right)\\ \cdot P\left(Alcohol\;intake\right)\cdot P\left(Coping\;style\right)\cdot P\left(Education\right)\;\cdot P\left(Age\right)\cdot P\left(Smoke\right)\cdot P\left(Insomnia\right)\\ \cdot P\left(Depression|Insomnia\right)\cdot P\left(Sleep1|Insomnia,Depression\right)\cdot P\left(Arthritis|Depression\right)\\ \cdot P\left(BMI|Smoke,\;Arthritis\right)\cdot P\left(Sleep2|Depression,Sleep1\right)\\ \cdot P\left(QOLp|BMI,\;Sleep2,Arthritis\right)\cdot P\left(QOLm|Depression,\;Sleep2\right)\cdot P\left(Insulin|BMI\right)\\ \cdot P\left(CRP|BMI\right)\\ \cdot P(PA|Age,\;Sleep2,\;Insulin) \end{array}$$ (2)

This decomposition converts the complex model comprising 19 variables into simpler components. It highlights subsets of factors that directly influence each variable. In fact, the maximum number of directed edges pointing to any variable is 3 (e.g., PA and QOLp), substantially fewer than the maximum of 18 possible directed edges. A first notable finding is that there were no edges from (or to) the following variables: tumor stage, years from diagnosis to study entry, neighborhood, education, alcohol intake and coping style (MB scale), indicating that these variables were (marginally) independent of all other factors. Below, we provide additional details on these decompositions, and how to infer (in)dependencies between variables.

### BMI and physical activity (PA)

The network allows us to elicit local structure of the variables and identify “parent” variables that directly influence other variables. In our learned network (Fig 1), the variables *smoke* and *arthritis* were parents of BMI. Table 2 provides parameter estimates, and strength of network links based on bootstrap analysis. The *smoke* and *arthritis* links to BMI were not very stable as reflected in the low arc-strengths from the bootstrap analysis: 0.63 for the *smoke*-*BMI* link, 0.54 for the *arthritis-BMI* link (Table 2). The regression coefficient for *arthritis* was positive, indicating that having arthritis was associated with higher BMI on average. Interestingly, the regression coefficient for *smoke* was positive as well, indicating that smoking was associated with higher BMI, which is contrary to the common belief that smoking can cause weight loss by suppressing appetite. However, smoking status in our cohort refers to “ever smoking” and likely reflects former smokers who quit many years ago; the proportion of current smokers in the cohort was <2%, too few to include as a separate variable in our analysis. In addition, BMI had a large Markov blanket comprising *smoke*, *sleep2*, *QOLp*, *arthritis*, *insulin*, *and CRP*, indicating its influence on multiple factors. Also, using Bayesian network theory, we can infer that BMI is independent of all other variables conditional on its Markov blanket.

**Table 2:** Parameter estimates and stability of a Bayesian network of total physical activity, sleep and other lifestyle factors, biomarkers, and physical and mental health outcomes in the Reach for Health cohort of 333 postmenopausal breast cancer survivors.

Outcome (child)	Predictors (parents)	Strength	Direction	Regression coefficients (SE)
Arthritis*	Depression	0.69	0.64	0.704 (0.239)
BMI	Smoke	0.63	1.00	1.157 (0.551)
	Arthritis	0.54	0.98	1.524 (0.555)
CRP	BMI	1.00	0.96	0.093 (0.012)
Depression*	Insomnia	0.93	0.60	1.000 (0.257)
Insulin	BMI	1.00	0.82	0.039 (0.006)
PA	Age	0.97	1.00	-4.563 (0.845)
	Sleep2	0.53	0.80	-1.905 (0.659)
	Insulin	0.73	0.77	-43.926 (11.188)
QOLm	Depression	0.95	0.97	-7.029 (1.717)
	Sleep2	1.00	0.95	-1.060 (0.094)
QOLp	BMI	0.83	1.00	-0.689 (0.176)
	Sleep2	1.00	1.00	-0.921 (0.096)
	Arthritis	0.80	1.00	-7.439 (1.740)
Sleep1^+^	Insomnia	1.00	0.97	10.641 (0.915)
	Depression	0.53	0.83	1.710 (0.845)

*: coefficients represent log-odds ratios

+: sleep1 = sleep disturbance, sleep2 = sleep impairment

Next, we examined links to PA. *Age*, sleep impairment (*sleep2*), and *insulin* level were parents of *PA (*Fig 1*)*. The association between *age* and *PA* had the highest arc strength (0.97), with a negative regression coefficient showing that higher age was associated with, on average, lower level of physical activity. The link between *insulin* and *PA* had a moderately high arc strength (0.73) and directional strength (0.77), whereas the link between *sleep2* and *PA* was weak (arc strength = 0.53) but had a relatively strong directional strength (0.80). The regression coefficients for *insulin* and *sleep2* were negative, implying that higher insulin level and poor sleep were associated with lower physical activity level. Since PA did not have any children in our learned network, the parents of PA also comprised the Markov blanket of *PA*. Thus, once we observe a subject’s *age*, *sleep2*, and *insulin*, her physical activity level is independent of all other variables in the network. It is interesting to note that *BMI* had a strong positive association with *insulin* (arc strength = 1.00, directional strength = 0.82). Hence, we can infer that BMI was indirectly negatively associated with PA.

### Biomarkers (insulin and CRP)

We are also interested in studying the local network structure of the two biomarkers, *insulin* and *CRP*. Both markers shared a single parent, *BMI*, and for both, this link appeared in 100% of the bootstrapped networks (arc strength = 1.00). The link between BMI and CRP also had very strong directional strength of 0.96, with a moderately high value of 0.82 for the BMI-insulin link. Both regression coefficients were positive, so that higher BMI was associated with higher insulin and CRP. The Markov blanket for *insulin* consisted of BMI, *age*, sleep2, and *PA*; and the Markov blanket for *CRP* only had only one element, *BMI*.

### Quality of life (physical and mental)

We also briefly summarize interesting associations revolving around physical and mental quality of life (*QOLp* & *QOLm*). *BMI*, *sleep2* and *arthritis* were parents of *QOLp (*Fig 1*)*. Both *BMI* and *arthritis* had strong associations with *QOLp*, with arc strength of 0.83 and 0.80 respectively. Regression coefficients showed that both *BMI* and *arthritis* were, as expected, negatively associated with QoLp *(*Table 2). We note that *arthritis* was directly, and indirectly via *BMI*, linked to *QOLp*, implying that *BMI* could be a mediator between *arthritis* and *QOLp*. Surprisingly, *sleep2* had the strongest association with *QOLp* (arc strength = 1.00), with a corresponding negative regression coefficient indicating that poor sleep quality was associated with worse physical quality of life.

*Depression* and *sleep2* were parents of *QOLm*, with respective arc strengths of 0.95 and 1, indicating that this cluster was strongly linked and highly reproducible. Again, as expected, the negative regression coefficients suggested that poor sleep and depression were associated with poorer mental QoL. Finally, via Markov blankets we infer that, conditional on *BMI*, *sleep2*, and *arthritis*, *QOLp* was independent of all other factors; and, conditional on *depression* and *sleep2*, *QOLm* was independent of all other variables.

### Hubs and subnetworks

In our network (Fig 1), the set (or any subset) of variables {insomnia, depression, QOLm} was conditionally independent of the set (or any subset) of variables {BMI, PA, QOLp, insulin, CRP}, given {sleep2, arthritis}. We point out that arthritis was in the set of conditional variables due to its link to depression, however, the arthritis-depression link was in fact weak with arc strength of 0.69 and even weaker directional strength of 0.64. This implies that sleep quality was the primary hub linking mental factors to physical health and biomarkers.

### Comparing networks

Given that sleep played a central role in our networks, we conducted network comparison analyses to test the importance of the two sleep quality measurements, *sleep1* and *sleep2*. We quantitatively assessed this assumption via Bayesian information criteria (BIC) scores. The original learned network had a BIC score of -14483.5 We then fit a second network by isolating *sleep1*, i.e., removing all links to and from *sleep1*, and obtained a BIC score of -14637.4, a 154-point lower score, indicating substantially worse fit for the model with the *sleep1* variable isolated compared to the original network. The Bayes factor for the original vs second model was approximately exp(-14483.5+14637.4), indicating > 20-fold higher posterior probability for the original network compared to the sleep-omitted network, thus affirming our hypothesis that *sleep1* plays a critical role in the network. Similarly, isolating *sleep2* resulted in an even larger reduction of 190 points in the BIC score, and hence a Bayes factor that strongly favored the original model. These analyses confirm the role of sleep as an important factor in the fitted network.

Given our focus on *BMI* and biomarkers, we conducted additional network comparison analyses to test the value of the learned sub-networks for *BMI* and the two biomarkers. We created a new network in which the edge from *BMI* to *insulin* was removed. The BIC score for this network was 20 points lower than that of the original network, and, as before, the Bayes factor would strongly favor the original model. Similarly, a network in which the edge from *BMI* to *CRP* was removed had a 26 points lower BIC score, again strongly favoring the original fitted model.

Finally, we investigated the depression-arthritis link, which was reproduced in only 69% of bootstrapped networks. Omitting this link, decreased the BIC score by 1.56, indicating only moderate evidence for this association.

### Deconstructing total PA

**Fig 2:**
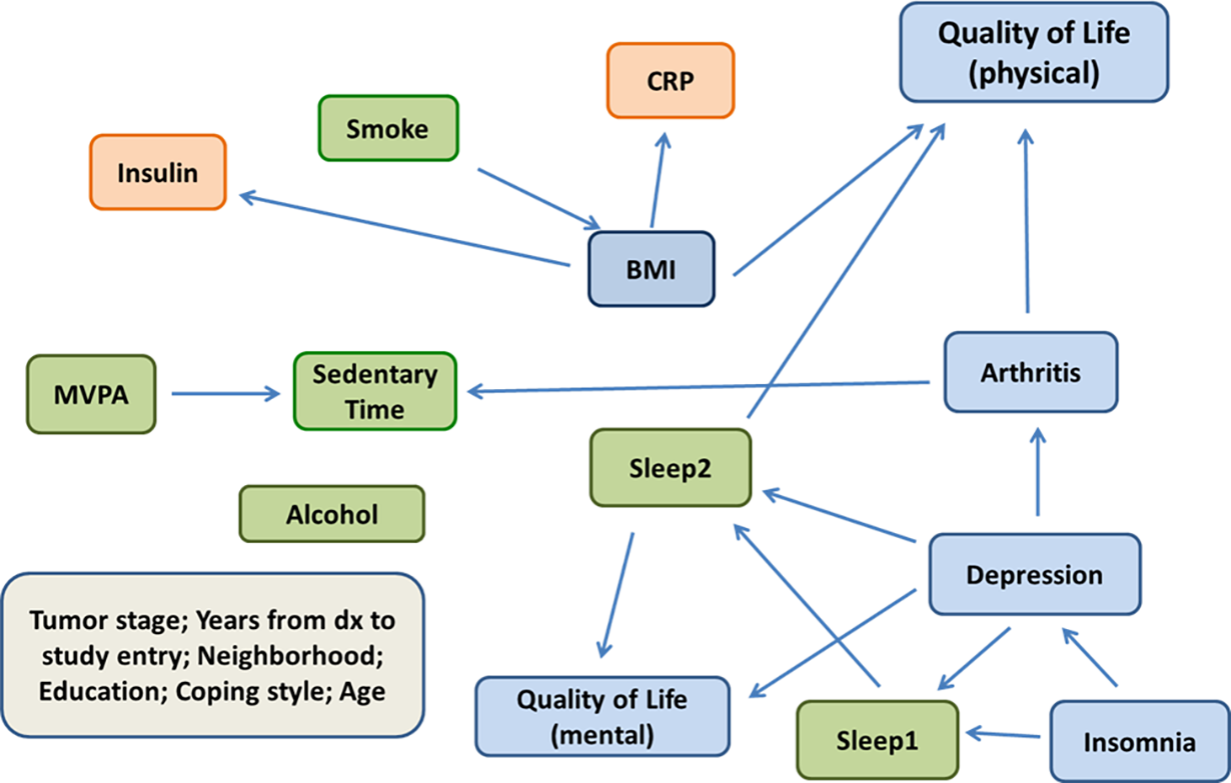
Bayesian network of moderate-vigorous physical activity, sedentary time, other lifestyle factors and health in the Reach for Health cohort. Lifestyle factors (green), biomarkers (orange), and physical and mental health outcomes (blue).

To further investigate physical activity, we parsed the total PA volume (counts/minute) variable as two activity behaviors: sedentary time and MVPA. When these two “activity” variables were included in the network instead of total PA, the network structure, i.e., parent and child nodes (Fig 2), were identical, and parameter estimates (Table 3) very similar to the original network for CRP, alcohol, smoking, sleep1, education, QoLm, QoLp, BMI, depression and insomnia. Two links were omitted: the arthritis-BMI edge and the age-PA edge, so that age was isolated and independent of all variables. With regards to activity, MVPA and arthritis were both direct parents, as well as, the Markov blanket of sedentary time, with lower MVPA and having arthritis associated with more sedentary time. The MVPA-sedentary time link was reproduced in 100% of the bootstrapped networks, and the network in which this link was omitted had a 28 point lower BIC score. The arthritis-sedentary time link was less robust occurring in 63% of bootstrapped networks with a corresponding 2.16 lower BIC score when this link was dropped.

**Table 3:** Parameter estimates and stability of a Bayesian network of moderate-vigorous physical activity, sedentary time, sleep, and other lifestyle factors, biomarkers, and physical and mental health outcomes in the Reach for Health cohort of 333 postmenopausal breast cancer survivors.

Outcome (child)	Predictors (parents)	Strength	Direction	Regression coefficients (SE)
Arthritis	Depression	0.69	0.62	0.704 (0.239)
BMI	Smoke	0.66	1.00	1.196 (0.557)
CRP	BMI	1.00	0.95	0.093 (0.012)
Depression	Insomnia	0.94	0.65	1.000 (0.257)
Insulin	BMI	1.00	0.85	0.039 (0.006)
QOLm	Depression	0.93	0.97	-7.029 (1.717)
	Sleep2	1.00	0.97	-1.060 (0.094)
QOLp	BMI	0.84	1.00	-0.689 (0.176)
	Sleep2	1.00	1.00	-0.921 (0.096)
	Arthritis	0.79	1.00	-7.439 (1.740)
Sedentary	Arthritis	0.67	0.99	-11.706 (7.178)
	MVPA	1.00	0.90	-1.788 (0.213)
Sleep1	Insomnia	1.00	0.96	10.641 (0.915)
	Depression	0.53	0.79	1.710 (0.845)
Sleep2	Depression	0.83	0.96	3.489 (0.741)
	Sleep1	1.00	0.93	0.665 (0.042)

### Predicting intervention effects

Edges and paths inferred from a Bayesian network can be used for prediction. If we perturb a node, e.g. PA or BMI, we can investigate predicted downstream effects on biomarkers. Table 4 gives a few examples of such queries: increasing total PA from <270 count/min/day to > = 380 counts/min/day (e.g., a PA increase of 1 SD) would be predicted to result in an average BMI reduction of 1 kg/m^2^. Or, moving from the obese to overweight category would be predicted to reduce insulin by 26% (reduction in loginsulin is 0.3pg/mL), reduce CRP by over 50% (reduction of 0.76 mg/L in logCRP), improve physical QoL by an average 6 points, and not change mental QoL appreciably. Most interestingly, reducing sleep impairment from the highest to the lowest quartile would be predicted to improve both mental and physical QoL by > 20 points. A combined change of reducing BMI category from obese to overweight *and* reducing sleep impairment from highest to lowest quartile would be predicted to result in a 26.7-point higher physical QoL score on average, suggesting that an intervention aimed at weight-loss and reducing sleep impairment could have additive effects on physical QoL. Importantly, the simulations also give estimates of variability in the outcomes corresponding to changes in the targeted behaviors (SDs in Table 4), which would be useful when estimating required sample sizes for intervention studies.

**Table 4:** Bayesian network propagation: Predicting change in outcomes.

Targeted behavior	Change in targeted behavior(s)	Outcome	Average change in Outcome [Mean(SD) of outcome by targeted behavior category]
PA	< 270 to ≥ 380 counts/min/day	BMI	BMI decreases 1 kg/m^2^ [31.5(4.9) to 30.5(4.9)]
BMI	≥ 30 to < 30 kg/m^2^	Insulin	Insulin decreases 0.30 (log) pg/mL (26% decrease) [6.29(0.50) to 5.99(0.49)]
BMI	≥ 30 to < 30 kg/m^2^	CRP	CRP decreases 0.76 (log) mg/L (50% decrease) [15.28(1.07) to 14.56(1.05)]
BMI	≥ 30 to < 30 kg/m^2^	QoLp	QoLp increases 6.1 [64.6(17.9) to 70.7(17.7)]
BMI	≥ 30 to < 30 kg/m^2^	QoLm	QoLm increases 0.3 [74.9(17.3) to 75.2(16,9)]
Sleep2	> Q3 to < Q1	QoLp	QoLp increases 20.5 [56.6(16.7) to 77.1(16.3)]
Sleep2	> Q3 to < Q1	QoLm	QoLm increases 23.9 [62.9(15.9) to 86.8(14.7)]
Sleep2 + BMI	sleep2 > Q3 to < Q1 BMI ≥ 30 to < 30 kg/m^2^	QoLp	QoLp increases 26.7 [54.3(16.1) to 81.0(16.1)\
Sleep2 + BMI	sleep2 > Q3 to < Q1 BMI ≥ 30 to < 30 kg/m^2^	QoLm	QoLm increases 23.3 [62.8(15.8) to 86.1(14.5)]

^a^ Derived from 5000 simulated datasets via network propogation.

Q3: 75^th^%-ile; Q1: 25^th^%-ile

## Discussion

In this work, we have illustrated how Bayesian networks, a machine learning tool, can be applied in behavioral research. Health behaviors are modifiable risk factors, and hence can be potentially intervened upon to improve health and reduce disease. Identifying which behaviors are most robustly linked to disease is critical for designing effective interventions, as changes in these will elicit the most robust health benefits. Bayesian networks can shed light on these solutions, as we enumerate below.

1. Identifying intervention targets: Bayesian networks provide insights into which factors directly affect health. For instance, in our analysis, BMI was directly linked to the biomarkers, suggesting that a weight-loss intervention could improve profiles of these markers. Similarly, sleep impairment was directly linked to Quality of life (mental and physical) suggesting that an intervention aimed at improving sleep quality could improve QoL. Of note, our network also suggests that a combined sleep improvement and weight-loss intervention could improve physical *and* mental QoL, as well as, glucose regulation and inflammation.
2. Mechanisms: Bayesian networks can identify indirect pathways of influence. For example, the arthritis-BMI-QoLp link indicates that high BMI is one of the mechanisms by which arthritis impacts QoL. Similarly, the depression-sleep2-QoLm link identifies sleep impairment as an intermediate factor by which depression impacts mental health. Again, these indirect paths through health behaviors suggest intervention targets, namely weight and sleep2, that could reduce the impact of arthritis and depression on physical and mental QoL respectively.
3. Informing study design: As shown in Table 4, Bayesian networks can be used to estimate putative intervention effects, and hence inform achievable effect-sizes and required sample-size.
4. Tailoring interventions: Bayesian networks can be useful for identifying at-risk populations and personalizing interventions. For instance, our network indicates that older age, more sleep impairment and higher BMI are each associated with lower physical activity, suggesting that these three factors could be used to custom design a physical activity intervention that will best suit the needs of specific subgroups.

We have enumerated a few ways in which Bayesian network analyses could inform public health research. The strengths of this work include a well-characterized cohort of breast cancer survivors, the availability of clinical information from medical records, objective information on physical activity, biomarker outcomes, and from a methodological perspective, the use of bootstrap methods and Bayesian information criteria, which reduce overfit and improve replicability. There are also limitations. Bayesian networks are an inherently exploratory tool, best suited for hypothesis generation. As mentioned before, the Markov blanket of a given node V identifies factors that directly influence V, and thus would be consonant with a causal (mechanistic) model. While it is impossible to prove causality from observational data, these methods can provide clues for particular causal models that would then have to be validated in experimental data and/or randomized trials. Hence our results need to be confirmed in other cohorts and/or randomized trials. Also, our cohort only included overweight postmenopausal cancer survivors who agreed to participate in an intervention trial, which could limit generalizability. For instance, it is possible that with an unrestricted BMI range, we may have observed other factors (e.g., built environment, age, PA) influencing BMI and other outcomes. Nevertheless, it would be interesting to test our final averaged network on younger and/or normal-weight breast cancer survivors.

In conclusion, we have introduced Bayesian networks, a machine learning methodology, to infer biobehavioral networks in a breast cancer cohort. Our results identified several health behaviors directly linked to biomarker and quality of life, suggesting potential mechanistic pathways and intervention targets. The network comparison analysis strongly favored the fitted networks, indicating that our findings are robust against alternative network structures. We believe that this network methodology could be a useful tool in health behaviors research.

## Supporting information

S1 Table Anonymized partial Reach for Health cohort participant information. (CSV)
